# Spatially resolved TiO_x_ phases in switched RRAM devices using soft X-ray spectromicroscopy

**DOI:** 10.1038/srep21525

**Published:** 2016-02-19

**Authors:** D. Carta, A. P. Hitchcock, P. Guttmann, A. Regoutz, A. Khiat, A. Serb, I. Gupta, T. Prodromakis

**Affiliations:** 1 Nano Group, Nanofabrication Centre, Electronics and Computer Science, Faculty of Physical Sciences and Engineering, University of Southampton, United Kingdom; 2 Chemistry and Chemical Biology and Brockhouse Institute for Materials Research, McMaster University, L8S4M1 Hamilton, ON, Canada; 3 Helmholtz-Zentrum Berlin für Materialien und Energie, Institute for Soft Matter and Functional Materials, Albert Einstein-Str. 15, 12489 Berlin, Germany

## Abstract

Reduction in metal-oxide thin films has been suggested as the key mechanism responsible for forming conductive phases within solid-state memory devices, enabling their resistive switching capacity. The quantitative spatial identification of such conductive regions is a daunting task, particularly for metal-oxides capable of exhibiting multiple phases as in the case of TiO_x_. Here, we spatially resolve and chemically characterize distinct TiO_x_ phases in localized regions of a TiO_x_–based memristive device by combining full-field transmission X-ray microscopy with soft X-ray spectroscopic analysis that is performed on lamella samples. We particularly show that electrically pre-switched devices in low-resistive states comprise reduced disordered phases with O/Ti ratios around 1.37 that aggregate in a ~100 nm highly localized region electrically conducting the top and bottom electrodes of the devices. We have also identified crystalline rutile and orthorhombic-like TiO_2_ phases in the region adjacent to the main reduced area, suggesting that the temperature increases locally up to 1000 K, validating the role of Joule heating in resistive switching. Contrary to previous studies, our approach enables to simultaneously investigate morphological and chemical changes in a quantitative manner without incurring difficulties imposed by interpretation of electron diffraction patterns acquired via conventional electron microscopy techniques.

Nanoscale resistive random-access memory devices (RRAM) based on metal-oxides, also known as memristors, have great prospect in becoming a mainstream memory technology, due to their infinitesimal dimensions, fast switching and capacity to store multiple bits of information per element^1^,^2^,^3^,^4^,^5^,^6^. Such devices have the ability to toggle their resistance between high resistive state (HRS, or ON) and low resistive state (LRS, or OFF) in either a digital or analogue fashion^7^. TaO_x_^8^ and HfO_x_^9^ are of particular interest for RRAM due to their high-*k* properties and compatibility with deep submicron complementary metal-oxide semiconductors (CMOS) technologies and their simple reduction dynamics; the ability to toggle between two phases only, makes them easy to control while at the same time enhances their state stability. On the other hand, TiO_x_, one of the most celebrated metal oxides, provides wider opportunities for multi-state memory capacity due to the intrinsic variety of possible chemical phases. This complexity however makes its control more difficult, also prohibiting the rigorous study of the unrelying physical mechanisms. Whilst the electrical behaviour of TiO_x_ based memristors has been widely investigated, much less is known about the atomic-scale changes that occur in the film as a consequence of switching^10^. It has been suggested that the formation and migration of defects within the oxide active layer induce the formation of reduced phases in form of conductive filaments that render resistive switching. However, direct localization and chemical characterization of such conductive phases remains a challenge^11^.

Previous work by Kwon *et al.*^12^ based on high resolution transmission electron microscopy (HRTEM), identified Ti_4_O_7_ as the structure of conductive filaments in TiO_2_ based-devices by interpretation of electron diffraction patterns. However, HRTEM requires the use of high energy electron beam (200–300 kV) which could induce sample crystallization and effectively alter the physical-chemical state of the film. Furthermore, electron diffraction requires the crystals to be aligned in specific orientations with respect to the beam for unambiguously capturing distinct diffraction patterns^12^. In this work, we employed soft X-ray spectromicroscopy for measuring Near-Edge X-ray Absorption Fine Structure (NEXAFS) spectra at high spatial resolution in a full-field Transmission X-ray Microscope (TXM). This technique allowed us to perform simultaneously imaging and spectroscopy for investigating morphological changes in the film as well as performing chemical analysis at nanometric scale of localized regions, respectively. Our set-up differs substantially from previous transmission X-ray microscopy studies of TiO_2_-based^13^,^14^ or SrTiO_3_-based^15^ RRAM devices, which are based on irradiating the device from the top electrode, alleviating the challenges imposed for accessing the TiO_x_ film through the capping top electrode. When compared to electron energy loss spectroscopy (EELS), TXM-NEXAFS provides more analytically useful information per unit radiation damage^16^. Moreover, identification of distinct phases using NEXAFS is based on fingerprints methods, avoiding the difficulties imposed via multiple scattering effects and/or the presence of diffraction spots from other TiO_2_ phases and/or metallic electrodes when employing electron diffraction.

This technique has allowed us to spatially resolve distinct TiO_x_ phases that macroscopically assemble in a conductive localized area within a RRAM prototype that has been switched to a LRS. Presumably, this conductive spot can be regarded as a conductive filament. However, as reversibility of switching in this region cannot be proved after cutting the lamella for the experiment, dielectric breakdown or Joule heating could also be responsible for the formation of the reduced area. We prove that the conductive path is located right underneath a protrusion of the top electrode and it is composed by a reduced TiO_x_ phase with a O/Ti ratio close to 1.37. Crystalline TiO_2_ rutile phase and an additional orthorhombic-like TiO_2_ phase regions were also identified in a localized area between the TiO_x_ reduced phase and the top electrode, suggesting that the temperature due to Joule heating increases up to 1000 K in this area^17^. A Ti-containing phase similar to the TiO_x_ reduced phase was also identified in both the top and bottom Pt electrodes^18^.

## Results and Discussion

**Figure 1:**
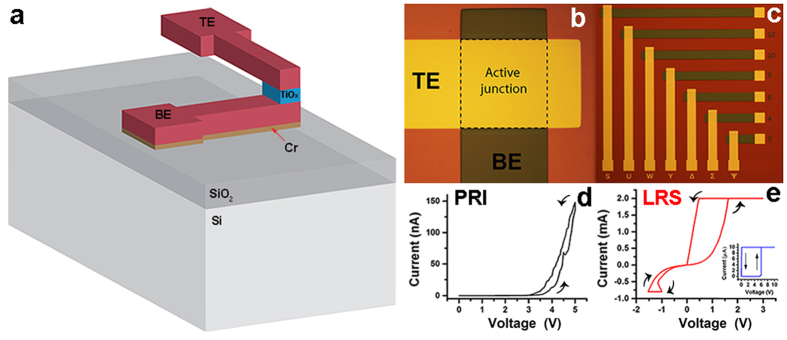
Device architecture and electrical characterisation of solid-state RRAM devices. (**a**) Schematic of the device stack structure. (**b,c**) Optical images of TiO_x_ RRAM stand-alone devices. (**d,e**) Electrical characterization of devices before TXM measurement: (**d**) I-V curve of pristine device showing an initial high resistive state; (**e**) LRS state device (Inset: electroforming step).

The studied prototypes are Cr(3 nm)/Pt(30 nm)/TiO_x_(50 nm)/Pt(30 nm) stacks (Fig. 1a), fabricated as stand-alone devices with a junction of 40 × 40 μm^2^ (Fig. 1b,c) (see Methods). The as-deposited TiO_x_ film was determined to be slightly reduced by X-ray photoelectron spectroscopy (Supplementary, Fig. S1). Prior the TXM-NEXAFS investigation, all devices were electrically characterized using voltage sweeping. In Fig. 1d, the current-voltage (I-V) characteristic of a pristine state device (PRI) is shown; note the extremely low conductance level. The device is subsequently electroformed (inset of Fig. 1e) before being switched to LRS (Fig. 1e main).

**Figure 2:**
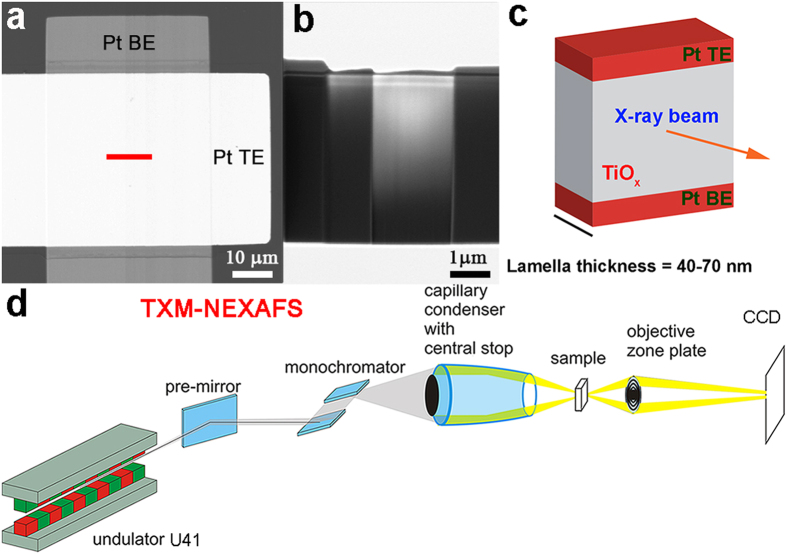
Experimental set-up. (**a**) SEM image of pristine device active junction. (**b**) SEM images of final thin lamella. (**c**) Lamella orientation for the TXM-NEXAFS experiment. (**d**) Schematic of the TXM for NEXAFS experiment.

TXM-NEXAFS measurements were performed on a cross-section of each of the two devices cases, *i.e.* PRI and LRS. An outline of the method is briefly described in Fig. 2. The lamella is extracted from the red region highlighted in Fig. 2a using the Focused Ion Beam (FIB) lift-out technique (see Methods). The thin lamella (Fig. 2b) is positioned perpendicular to the X-ray beam direction for absorption measurements and imaging as shown in Fig. 2c. A schematic of the TXM experiment is shown in Fig. 2d.

For each of the two cases, PRI and LRS, two sequences (stacks) of X-ray images were acquired at closely spaced photon energies using a transmission X-ray microscope, in the Ti 2*p* (450–485 eV) and O 1 *s* (525–555 eV) energy range. Each stack, consisting of 1053 images over the Ti 2*p* energy range and 351 images over the O 1 *s* energy range, was carefully aligned using a cross-correlation iteration process until the image shift was less than ±0.6 pixels (±3 nm) across the entire energy range (see Methods). After conversion of all stacks into optical density (absorbance), a detailed analysis involving extraction of the X-ray absorption spectra of specific regions of interest and conversion to component maps using spectral fitting or multivariate statistical analysis procedures was performed^19^,^20^.

**Figure 3:**
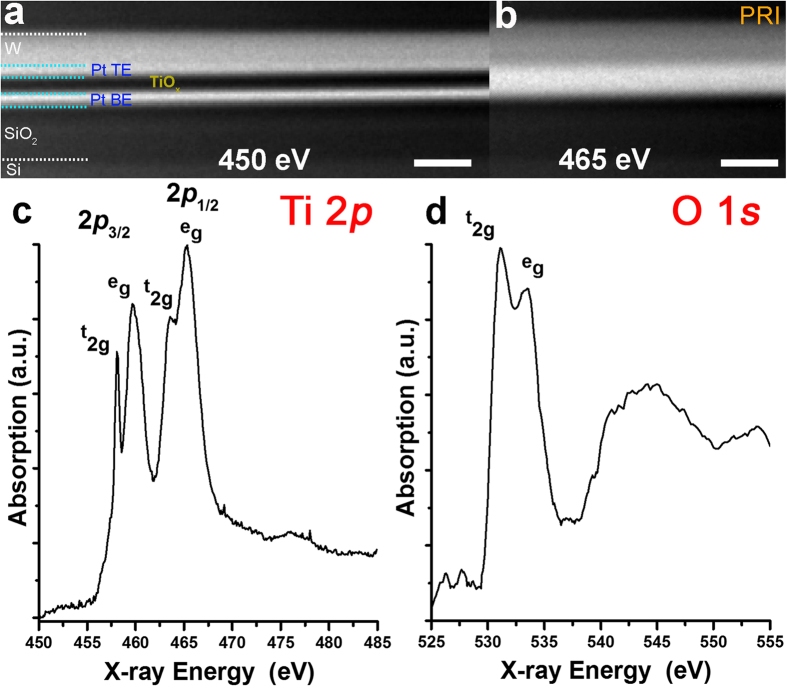
Optical density TXM micrographs of the PRI case obtained using X-ray photon energy of 450 eV (**a**) and 465 eV (**b**) and Ti 2*p* (**c**) and O 1 *s* (**d**) NEXAFS spectra extracted from the TiO_x_ film. Scale bar = 200 nm.

X-ray images of the lamellae for the PRI case at 450 eV (below the Ti 2*p* edge) and 465 eV (on the strongest Ti 2*p* absorption peak) are presented in Fig. 3. The individual layers comprising the device stack *i.e.* Si wafer, SiO_2,_ Pt bottom electrode (BE), TiO_x_ active film and Pt top electrode (TE) can be clearly distinguished in Fig. 3a; the TiO_x_ active layer appears dark as Ti does not absorb at this energy. At 465 eV, the TiO_x_ layer appears bright due to the strong absorption of Ti (Fig. 3b). Spatially localized NEXAFS spectra at the Ti 2*p* and at the O 1 *s* were then extracted from the TiO_x_ film area. Two representative spectra are reported in Fig. 3c,d, respectively. The fine structure of Ti 2*p* spectrum is complex but at a coarse level can be considered to consist of four main peaks (two doublets). The first doublet (2*p*_3/2_) occurs within 457–462 eV while the second doublet (2*p*_1/2_) appears in the range 462–468 eV (see Supplementary, TXM-NEXAFS section for details)^21^. The Ti 2*p* spectrum exhibits distinct features for the different polymorphs phase of crystalline TiO_2_ and for amorphous TiO_2_^22^. In particular, peaks of amorphous TiO_2_ are quite broad due to structural disorder caused by a range of bond angles and lengths, in contrast to spectra of crystalline samples which are characterized by sharp peaks. The Ti 2*p* spectrum shown in Fig. 3c is consistent with amorphous TiO_x_^22^,^23^. This result is in agreement with a TiO_x_ thin film prepared via reactive sputtering and indicates damage/crystallisation was not caused by the sample preparation process. In agreement with our observations at the Ti 2*p*, the O 1 *s* spectrum shown in Fig. 3d, possesses typical signatures of disordered systems^22^, demonstrating that the TiO_x_ film is amorphous all along the cross-section.

**Figure 4:**
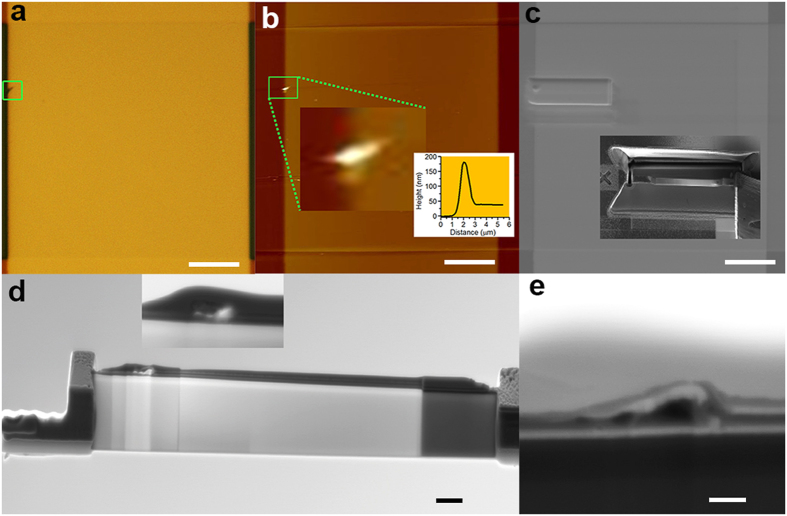
(**a**) Optical and (**b**) AFM images viewed from the TE. Inset in (**b**) line profile of the protrusion. (**c**) SEM image of lamella cut location and in inset detail of lamella extraction. (**d**) Thinned lamella with details of the damaged region. (**e**) Cross-section SEM image of the damaged region. Scale bar (**a–c**) 10 μm; (**d**) 1 μm; (**e**) 200 nm.

Notable differences are however observed in the film after switching to LRS (Fig. 4). Optical images (Fig. 4a and Supplementary, Fig. S2) and AFM images (Fig. 4b) viewed from the Pt TE show the formation of a protrusion about 140 nm high at the top left rim of the TE. Several studies have shown that protrusions of the TE could indicate possible critical regions of the film responsible for the RS switching^24^,^25^. The LRS lamella cut across the TE defect (Fig. 4c), presents some electron transparent areas in the damaged region (Fig. 4d) indicating the presence of a very low absorption area. Under the identified protrusion, the TE is bulged and discontinuous, having the shape of a dome (Fig. 4e). The region below this dome is formed by a main open cavity with two additional smaller ones towards the region outside the junction. The TiO_x_ layer is dislocated towards the region where the TE is discontinuous. The dome-like empty area suggests that the TE protrusion was caused by O_2_ gas escaping through the weakest point of the TE, the rim, in agreement with previous observations^24^,^25^. These features are similar to those reported in ref. 11.

**Figure 5:**
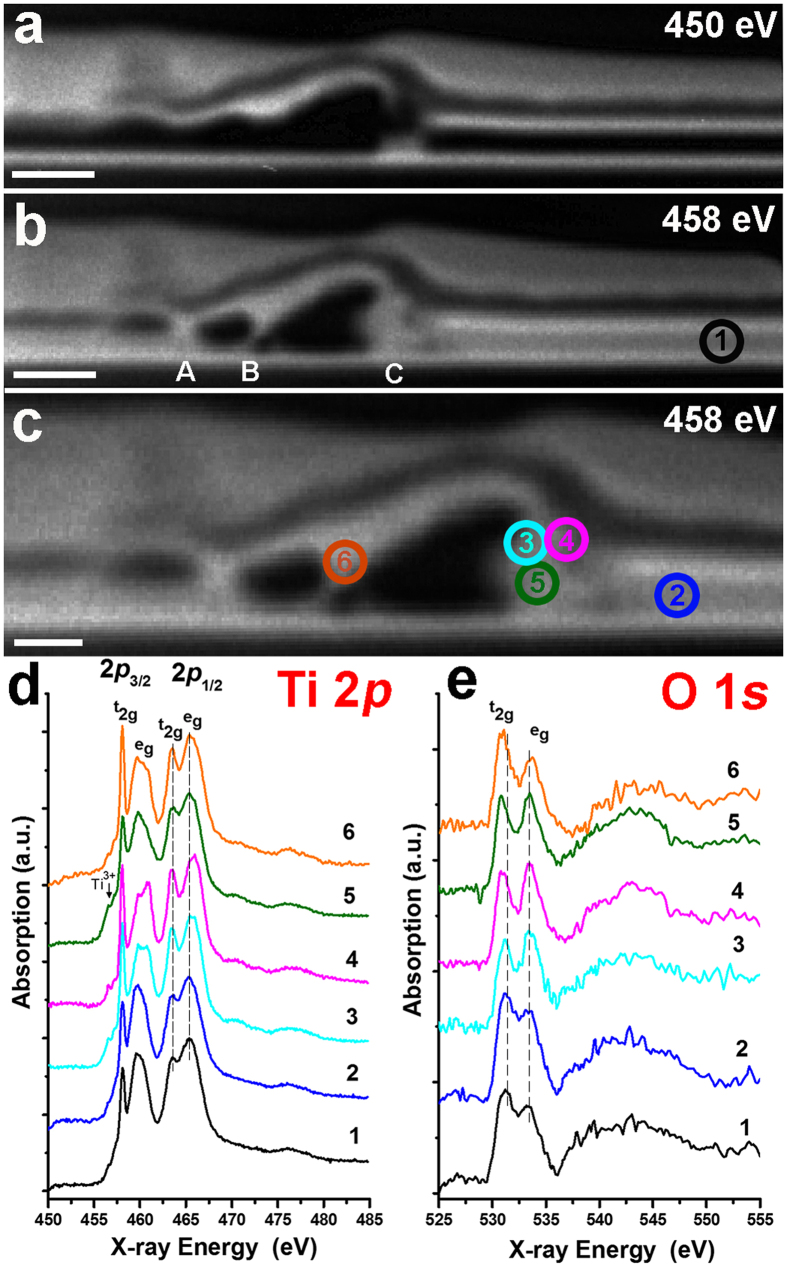
Physicochemical characterization of a RRAM device programmed in LRS. Optical density TXM micrographs of LRS device obtained with photon energy of 450 eV (**a**) and 458 eV (**b,c**). NEXAFS Ti 2*p* (**d**) and O 1 *s* (**e**) spectra extracted from the regions circled in the X-ray images b and c. Scale bar (**a,b**) 150 nm; (**c**) 100 nm.

The X-ray cross-section image of the LRS case recorded in the region underneath the TE protrusion at the energy of 450 eV is reported in Fig. 5a. The Pt/TiO_x_/Pt stack can be clearly distinguished on the right side of the image (Pt BE/TE are bright and TiO_x_ is black) and appear unaffected. In the defect region, the BE remains unaffected whereas the TE is discontinuous and delaminated from the TiO_x_ film. At 450 eV, the Ti containing regions cannot be distinguished from void zones as both appear dark. By examining the Ti 2*p* image sequence at different energies, we can spatially resolve the location of regions containing Ti species as the contrast of the image changes in correspondence with changes in the Ti absorption peak intensities. The image at 458 eV (Fig. 5b) shows highly absorbing regions containing TiO_x_ appearing with brighter contrast; the regions that appear dark at 450 eV but bright at 458 eV correspond to Ti containing areas. Regions that remain dark at 458 eV are clearly defined as voids. It is interesting to note that three highly localized (filamentary) Ti containing regions, (indicated as A, B and C – see Fig. 5b) link the BE and TE. In particular, the magnified image of Fig. 5c, shows that the TiO_x_ layer aggregates in a ~100 nm wide localized region underneath the highest deformation of the TE (C). These observations are consistent with the X-ray coloured image shown in Supplementary, Fig. S3 where the areas containing only Ti species are shown in red.

By extracting NEXAFS spectra in the regions where Ti species exist, chemical identification of the species can be performed as NEXAFS spectra are specific for each TiO_x_ phase. 6 regions of interest (ROIs) were identified, indicated in Fig. 5b,c by numbered circles. The Ti 2*p* and O 1 *s* X-ray absorption spectra extracted from each of these localized regions are reported in Fig. 5d,e, respectively. There are significant variations in the Ti 2*p* spectral shapes clearly indicating significant changes in the chemical structure of TiO_x_. Peak splitting and changes in relative intensities of the Ti 2*p* peaks can be attributed to changes in symmetry around Ti. In particular, the (2*p*_3/2,_ *e*_*g*_) orbitals at the Ti 2*p* point directly towards the 2*p* orbitals of the surrounding O and are therefore very sensitive to the titanium local environment (Fig. S4a)^22^,^26^.

These spectral differences were then employed to spatially resolve different TiO_x_ phases across the TiO_x_ film.

Spectra extracted from undamaged areas of the film far away (ROI_1) and close (ROI_2) to the identified defect, are similar to each other. They show broad and smooth spectral features, higher intensity of the (2*p*_3/2_, e_g_) peak compared to that of the (2*p*_3/2_, t_2g_) peak^27^ and absence of splitting of the (2*p*_3/2_, e_g_) peak around 460 eV; all these features indicate a high structural disorder as expected for amorphous TiO_x_^22^,^23^. The spectra extracted in the defect regions, ROI_3, ROI_4 and ROI_6, are quite different and characteristic of crystalline TiO_2_. A notable finding is the splitting of the (2*p*_3/2_, e_g_) peak, which indicates distortion of the TiO_6_ octahedra^28^. The relative intensities of these two peaks depend on the particular type of octahedral distortion, therefore on the particular polymorphic form of TiO_2_ (see Supplementary, Fig. S4a for details)^29^.

In the Ti 2*p* spectrum of ROI_3, the intensities of these two peaks are comparable. This particular (2*p*_3/2_, e_g_) profile has been reported for orthorhombic-like phases such as TiO_2_-II^30^ and brookite^31^. The profile of ROI_3 region is very similar to that of the TiO_2_-II phase reported in^30^, an usually high pressure phase which has recently been grown at ambient pressure by atomic layer deposition as 50 nm thin films^32^. In the spectrum of ROI_4, the relative intensities of the split (2*p*_3/2_, e_g_) peak are different, being higher for the peak at higher energy. This is typical of TiO_2_ rutile phase which has tetragonal distortion of the TiO_6_ octahedra^22^,^31^,^33^. Assignment of this spectrum to the rutile phase is also supported by the fact that the crystal-field splitting of the 2*p*_1/2_ peak is 2.4 eV^28^. Finally, in the spectrum of ROI_6, the relative intensity of the split (2*p*_3/2_, e_g_) peak is opposite to that in rutile, being the intensity higher for the peak at lower energy. This profile is typical of anatase. Moreover, the crystal-field splitting of the 2*p*_1/2_ peak is 1.9 eV, lower than the one observed in rutile, and typical of the anatase phase^30^,^28^. Two additional low intensity peaks appear in ROI_3, ROI_4 and ROI_6 at 456.6 and 457.2 eV (indicated as a and b in Supplementary, Fig. S4a) are also observed in rutile, anatase^22^ and TiO_2_-II^30^. They are atomic multiplet features related to exchange interaction between 3*d* electrons and the 2*p* core hole^21^ which tend to be very similar in different species, but may be modified in lower symmetry environments or when there are other interactions affecting the 3*d* partial density of states^34^.

The spectrum of ROI_5 is quite peculiar and represents the predominant species observed underneath the TE defect. The intensity of (2*p*_3/2_, t_2g_) peak in the Ti 2*p* spectrum extracted from ROI_5 is lower than that in the spectrum from ROI_3, ROI_4 and ROI_6, indicating lower local symmetry at point 5. The low energy shoulder around 456 eV is consistent with the presence of a partially reduced phase containing Ti^3+^ 13,27. This is also confirmed by the fact that the peaks are slightly broader compared to ROI_3, ROI_4 and ROI_6, as inferred from the reduced 2*p*_3/2_ and 2*p*_1/2_ peak to valley ratio (see Supplementary, Fig. S4a for details)^35^. Spectra of phases containing Ti^3+^ show broader features compared to the pure Ti^4+^ phases because of the binding energies of the Ti^3+^ levels and overlapping and increasing numbers of allowed transitions in the Ti^3+^ spectra^36^,^37^. Formation of Ti^3+^ species underneath the defect can be explained by the following reaction:


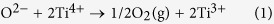


O_2_ gas accumulates below the TE and erupts from the weakest part of the film, causing the physical dome-like deformation observed at the rim of the TE^38^. The edge of the TE has been suggested recently to be the preferred reduction site since it is a three-phase contact site (oxide, metal and ambient atmosphere)^39^.

Formation of Ti^3+^ upon switching has been previously reported by Strachan *et al.*^13^. In this work, Scanning Transmission X-ray Microscopy (STXM) was used for phase identification of nanoscale channels underneath TE defects of Pt/TiO_2_/Pt devices. It is interesting to note that whereas the Ti 2*p* spectrum measured underneath a defect in a region near the centre of the device shows features similar to Ti_4_O_7_, the corresponding spectrum underneath a defect located at the rim of the top electrode of a LRS device, is quite similar to the one observed in the present work, with a low energy shoulder around 457 eV and a minor broadening of the 2p_3/2_ and 2p_1/2_ doublets.

The corresponding O 1 *s* X-ray absorption spectra extracted from the same localized regions used to extract the Ti 2*p* edge spectra are reported in Fig. 5e and Supplementary, Fig. S4b. Variations between the selected ROI_s are less evident at the O 1 *s* as the change in structure of TiO_x_ has less influence on O 1 *s* than Ti 2*p* spectra^40^. Nevertheless, our analysis of O 1 *s* spectra is in good agreement with that of Ti 2*p*. O 1 *s* spectra of undamaged areas ROI_2 (and the similar ROI_1) show broad and smooth spectral features typical of amorphous TiO_x_^23^. Moreover, the dip between the (O 1 *s*, t_2g_) and (O 1 *s*, e_g_) peaks is shallow compared to the spectra of regions from ROI_3 to ROI_6 and their energy difference, which is often used to evaluate crystal field splitting, is smaller and typical of disordered materials (2.0 eV)^26^. In the spectra extracted from ROI_3 to ROI_6, the e_g_ and t_2g_ peaks appear more defined and intense. The energy gap between e_g_ and t_2g_ in ROI_4 and ROI_6 is ~3.0 eV, a value reported for both anatase and rutile phases^22^. This gap is slightly smaller in ROI_3.

**Figure 6:**
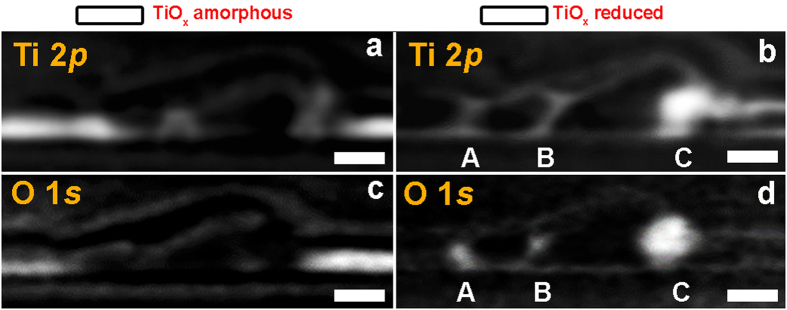
Chemical maps depicting regions of amorphous and reduced TiO_x_. Components maps at the Ti 2*p* (**a,b**) and at the O 1 *s* (**c,d**). Scale bar = 100 nm.

In order to better visualise the chemical distribution of the main TiO_x_ phases across the LRS case, the aligned Ti 2*p* and O 1 *s* image sequences on an optical density scale were converted to chemical components maps by means of singular value decomposition (SVD) procedures^41^. The black and white chemical maps at the Ti 2*p* (Fig. 6a,b) and O 1 *s* (Fig. 6c,d) were obtained using the two predominant components in the film, namely amorphous (corresponding to the spectrum of ROI_1 and ROI_2) and reduced (corresponding to the spectrum of ROI_5) TiO_x_ phases. The brightest regions correspond to TiO_x_ amorphous in Fig. 6a,c and TiO_x_ reduced in Fig. 6b,d. The fit of the two components gives consistent results at the Ti 2*p* and O 1 *s* edges. In the unaffected regions, TiO_x_ remains amorphous in between the electrodes whereas the reduced TiO_x_ phase is localized in a well-defined region underneath the TE protrusion (C) and in the two thinner filamentary regions connecting TE and BE on the left side of the protrusion (A and B).

**Figure 7:**
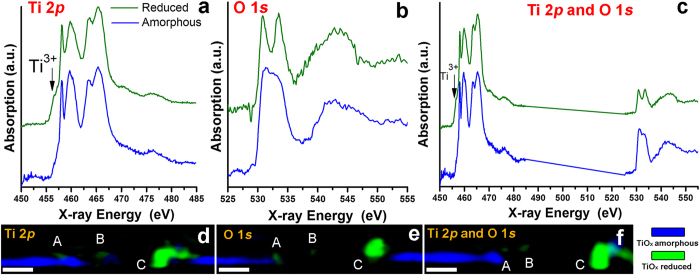
Physicochemical characterization of a RRAM device programmed in LRS . Color-coded composition maps at the Ti 2*p* and O 1 *s* generated independently (**d,e**) and simultaneously (**f**) by using the absorption spectra of the two components, TiO_x_ amorphous (blue) and TiO_x_ reduced (green) shown in (**a–c**). Scale bar = 100 nm.

The spatial correlation of amorphous and reduced TiO_x_ phases is better visualized by merging the two components in single color-coded composition maps. Spectra of these components at the Ti 2*p* and O 1 *s* are respectively shown in Fig. 7a,b and were used to generate the color-coded composition maps reported in Fig. 7d,e, with blue regions corresponding to amorphous and green to reduced TiO_x_.

Localization of the two phases is apparent with the TiO_x_ amorphous in the unaffected film areas whereas the reduced TiO_x_ is localized mainly below the highest protrusion of the defect (C) and in the regions A and B. As reduced TiO_x_ has increased conductivity compared to the amorphous state, this is a direct observation of the area responsible for the LRS state of the device. Our observations are further validated by appending the Ti 2*p* and O 1 *s* stacks of the same ROI area (Supplementary, Fig. S5) and by appending the Ti 2*p* and O 1 *s* spectra into a single combined spectrum for each of the amorphous and reduced TiO_x_ regions (Fig. 7c). The spatial distributions of the amorphous and reduced TiO_x_ regions shown in Fig. 7f are in excellent agreement with those derived from considerations of the Ti 2*p* or O 1 *s* edges independently, supporting the validity of our analysis and giving further evidence of the highly reduced TiO_x_ percolation.

Further analysis was performed by generating chemical components maps exploring various combinations of reference spectra extracted from the image sequences. For example, the colour-code composite map at the O 1 *s* shown in Supplementary, Fig. S6b, was obtained by including in the fitting the O 1 *s* spectrum of the SiO_2_ layer below the BE (Supplementary, Fig. S6a). A satisfactory final fit of the Pt(BE)/TiO_x_/Pt(TE) cross-section was obtained by adding a third component in addition to amorphous and reduced TiO_x_ (Supplementary, Fig. S7). The Ti 2*p* spectrum of the third component (represented in red in Supplementary, Fig. S7a and S7b) is similar to that of the reduced phase but exhibits a smaller 2*p*_1/2_ crystal field splitting and it has a significant lower intensity compared to the reduced TiO_x_. The O 1 *s* spectrum of the third component is quite different from that of reduced TiO_x_. Combined color-coded maps obtained independently at the Ti 2*p* and O 1 *s* (Supplementary, Fig. S7c and S7d), show that the third phase, represented in red, is clearly present only in the TE and BE regions. This also explains the observed lower intensity in comparison to the other two components. Combined fitting at Ti 2*p* and O 1 *s* further validates this finding (Supplementary, Fig. S7e). The identification of the phase observed in BE and TE is not straightforward. The O 1 *s* spectrum suggests the formation of a new phase, perhaps a Pt alloy. The presence of Ti in both Pt TE and BE could be caused by interdiffusion or by different bonding situations of Ti and O at the TiO_x_-electrodes interfaces. Other causes such as misalignment due to sample tilting or image shifting are very unlikely as careful alignment procedures (of sample and images) were performed before analysis (Supplementary, Fig. S8).

The combined approach allows us to estimate the O/Ti ratio of the three species used for the fitting by performing an atom-edge jump analysis (Supplementary, Table S1). Our results indicate that the O/Ti ratio is 1.85±0.15 for the amorphous film, 1.37 ± 0.15 for the reduced and 5±1 for the reduced phase in the Pt BE and TE. Chemical mapping results combined with point spectra analysis of selected ROI_s show that the core of the conductive region is formed by a reduced phase with a O/Ti ratio close to 1.37 adjacent to a crystalline phases such as TiO_2_ rutile and brookite or TiO_2_-II in the region between the reduced area and the delaminated TE area. It is also interesting to note that the profile of region ROI_3 is very close to that of orthorhombic TiO_2_-II which has been recently prepared by ALD^42^ and found to be an intermediate phase in rutile formation by ball milling^43^. Its presence could support the argument of local stress manifested due to the switching. The presence of crystalline phases adjacent to the reduced TiO_x_ area confirms that the current passes through the reduced localized area and the heating is mainly localized in this region. The increase in temperature necessary for the growth of crystalline TiO_2_ phases is induced by the localized Joule heating produced during the switching cycles. It was previously shown that by annealing a TiO_2_ amorphous film, the anatase phase is formed first because of energy surface minimization around 650 K^44^,^45^. Formation of rutile only occurs after further annealing at higher temperatures, around 1000 K^17^. In our film, the anatase phase is only observed in a region slightly away from the core of the conductive region, where a small reduced area is observed. However the rutile phase was identified in the region adjacent to the main reduced area. This suggests that the Joule heating temperature reached in the main conductive region as a consequence of the bias reaches 1000 K.

By recording a sequence of X-ray images of the cross-section of the TiO_x_ thin film at closely spaced photon energies, we have directly visualized the location of the reduced area and characterized it chemically at a very fine spatial scale by extracting Ti 2*p* (450–485 eV) and O 1 *s* (525–555 eV) X-ray absorption spectra. Our results demonstrate that the conductive localized region is composed by a reduced TiO_x_ phase with a O/Ti ratio close to 1.37 and that it is located right underneath a protrusion of the top electrode. Crystalline TiO_2_ rutile phase and an additional orthorhombic-like TiO_2_ phase regions were identified for the first time, in a localized area between the TiO_x_ reduced phase and the top electrode of the device. This provides the first experimental indication that the temperature in the core of the conductive region increases up to 1000 K. A Ti-containing phase similar to the TiO_x_ reduced phase was also identified in both the top and bottom Pt electrodes, giving evidence of Ti diffusion into the Pt contact electrodes due to the switching. This technique can be adapted for other RRAM materials, such as NiO^46^, Cu_2_O^47^ and VO_2_^48^, enabling more in-depth studies in reduction dynamics.

## Methods

### Device Preparation

The Cr/Pt/TiO_x_/Pt based memristor devices were fabricated on an oxidised (200 nm SiO_2_) 6-inch Si wafer. The bottom electrode (BE) and top electrode (TE) were fabricated using conventional optical lithography, electron-beam evaporation followed by a lift-off process, with BE and TE composed of Cr/Pt (3 nm/30 nm) and Pt (30 nm) successively. The Cr film in the BE served as adhesive layer for Pt. A 50 nm TiO_x_ layer was then deposited using reactive sputtering from a Ti metal target with the following settings: 8 sccm O_2_, 35 sccm Ar, 2 kW at the cathode, and 15 sccm O_2_ and 2 kW at the additional plasma source.

### Electrical switching of devices

Electrical biasing of the devices has been carried out through voltage sweeping in order to assess or modify their resistive states as required (2 V maximum). Passive resistive state assessment of the Device Under Test (DUT) was carried out via low voltage, non-invasive voltage sweeps whilst resistive switching was induced through the application of more aggressive, high voltage sweeps. All voltage biasing was carried out under current compliance protection. The compliance current level was determined on *ad hoc* basis. All sweeping was performed using a Keithley 4200 electrical characterisation instrument.

### FIB-SEM

A dual-beam focused ion beam/scanning electron microscope system (Zeiss NVision 40 FIB/FEGSEM) equipped with a gas injection system (GIS) was used to record SEM images and for cutting FIB cross-sections. SEM images were recorded at an accelerating voltage of 5 kV. Prior to performing FIB cross-sections, an electron beam-induced tungsten protective layer was deposited on the top of the electrodes in order to minimize damage caused by the gallium ions in the subsequent ion beam-induced tungsten deposition step. After extraction, the thickness of the lamella is further decreased to allow X-ray transmission by low energy ion polishing at a low incident angle until a thickness of 40–70 nm is achieved.

### TXM-NEXAFS

The TXM-NEXAFS study was performed at the undulator beamline U41-FSGM at the BESSY II electron storage ring operated by the Helmholtz-Zentrum Berlin, Germany. The beamline itself and also the environment of the sample is kept under high vacuum conditions. The optical design of TXM has a spatial resolution of 25 nm and spectral resolution E/ΔE = 10000^49^.

Samples alignment was checked using a test specimen with defined distances between structures employing the same sample holder used for the actual experiment. The alignment along the beam direction was done by having the correct focus for each photon energy. The Ti 2*p* images were acquired from 450 to 485 eV in 0.1 eV steps of photon energy. In order to increase the signal to noise ratio of Ti 2*p* spectra, three separate images were acquired for each photon energy giving a total of 1053 images. The O 1 *s* images were acquired from 525 to 540 eV in 0.1 eV steps and from 540 to 555 eV in 0.2 eV steps giving a total of 351 images. Images size is 1340 × 1300 pixels size corresponding to 5 nm per pixel. Each image was taken with the sample at the proper focus position to optimize spatial resolution^50^. First, a stack of all images was created followed by automatic alignment using Fourier cross correlation techniques in aXis2000 and in Stack_Analyze. Careful alignment of images was performed using the Zimba procedure to correct for lateral motion of the X-ray beam on the sample^50^. Alignment was iterated until the x,y deviations were less than 0. 6 pixels (±3 nm). The I_o_ for each stack was obtained from an internal region of each stack (off the FIB section) and used to convert the aligned stack from transmission to optical density (OD = −log[I(E)/Io(E)]). Ti 2*p* and O 1 *s* spectra were then extracted from the stack. Spectra can be obtained from regions as small as the spatial resolution of the microscope (25 nm). Data analysis was performed using software aXis2000^51^.

### AFM

AFM maps were acquired with a MultiMode Nanoscope V AFM (Veeco Metrology Group) in contact mode using Pt/Ir coated Si tips with a cantilever spring constant of 0.2 N/m and nominal radius of 12 nm (Bruker, SCM-PIC).

## Additional Information

**How to cite this article**: Carta, D. *et al.* Spatially resolved TiO_x_ phases in switched RRAM devices using soft X-ray spectromicroscopy. *Sci. Rep.* **6**, 21525; doi: 10.1038/srep21525 (2016).

## Supplementary Material

Supplementary Information
