# Supplementary material for: Spatially resolved TiOx phases in switched RRAM devices using soft X-ray spectromicroscopy
Source: Sci Rep. 2016 Feb 19;6:21525. doi: 10.1038/srep21525 (PMC4759601; doi:10.1038/srep21525)
Supplement: Supplementary Information [file srep21525-s1.pdf]

Supplementary Information

Spatially resolved TiO_x phases in switched RRAM devices using soft X-ray spectromicroscopy

D. Carta[a,*], A. P. Hitchcock[b], P. Guttmann[c] , A. Regoutz[a], A. Khiat[a], A. Serb[a], I. Gupta[a], T. Prodromakis[a]

[a] *Nano Group, Nanofabrication Centre, Electronics and Computer Science, Faculty of Physical Sciences and Engineering, University of Southampton, United Kingdom*

[b] *Chemistry and Chemical Biology and Brockhouse Institute for Materials Research, McMaster University, L8S4M1 Hamilton, ON, Canada*

[c] *Helmholtz-Zentrum Berlin für Materialien und Energie, Institute for Soft Matter and Functional Materials, Albert Einstein-Str. 15, 12489 Berlin, Germany*

* Corresponding author. E-mail: d.carta@soton.ac.uk, Tel: +44 (0)23 8059 3737, Fax: +44 (0)23 8059 3029

1. X-ray photoelectron spectroscopy (XPS)

XPS was used to characterise the TiO_x thin film. All spectra were recorded on a Thermo Scientific Theta Probe Angle-Resolved X-ray Photoelectron Spectrometer (ARXPS) system with a monochromated Al Kα X-ray source (hν = 1486.6 eV). The X-ray source was operated at 6.7 mA emission current and 15 kV anode bias and pass energies of 200 eV and 50 eV were used for survey and core level spectra, respectively. Spectra were corrected for any charge shifts by aligning them to the C 1*s* core level at 285.0 eV and all data were analysed using the Avantage software package.

The XPS survey spectrum of the TiO_x thin film along with the O 1*s* and Ti 2*p* core levels are shown in Fig. S1a, S1b and S1c, respectively. The Ti 2*p* core level shows charge transfer satellites $S_{3/2}$ and $S_{1/2}$ at higher binding energies. [1] Furthermore, a small population of Ti^{3+} of the order of 4 % of the total Ti is observed (Fig. S1d). The O 1*s* core level shows a surface oxygen component, often referred to as non-lattice oxygen, on the higher binding energy side of the main core line.[2]

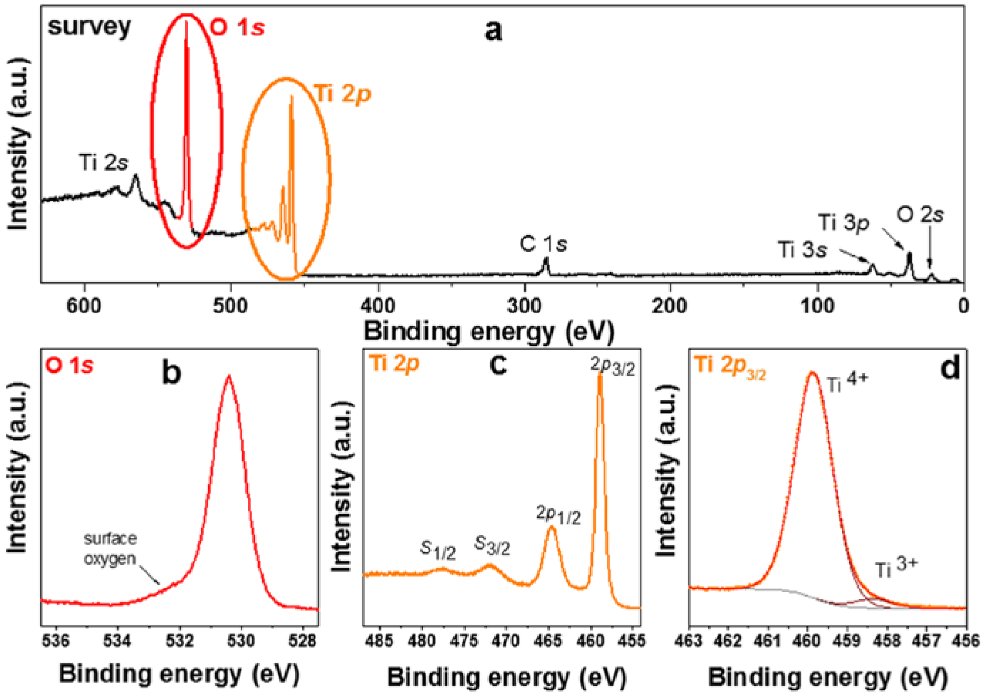

Figure S1 | XPS spectra of TiO_x. **(a)** Survey spectrum of TiO_x showing all core levels. **(b)** O 1*s* core level. **(c)** Ti 2*p* core level including higher binding energy satellites $S_{3/2}$ and $S_{1/2}$. **(d)** Fit of the Ti $2p_{3/2}$ peak.

2. TXM-NEXAFS

The TXM set-up used in this work presents significant advantages compared with previously used geometries. In particular, it does not require replacement of the silicon wafer support with a fragile standing Si_3N_4 window [3,4] (which could affect the device electrical behaviour due to strain effects and difficulty in sinking the Joule heating [5,6]) and does not require removal of the top electrode prior

to analysis (which is usually performed by a scotch tape method and could lead to unwanted removal of the thin TiO_2 layer underneath critical areas [5,7]), both necessary steps to enable the X-ray transmission if irradiating the device from the top electrode. Most importantly, our geometry allows direct visualization and chemical investigation of the cross-section of the device, as shown in Fig. 3a (main manuscript). Ti 2*p* and O 1*s* spectra extracted from the TiO_x film in PRI device case are shown in Fig. 3c and 3d (main manuscript), respectively. The first doublet ($2p_{3/2}$) (457-462 eV) of Ti 2*p* spectra (Fig. 3c) originates from transitions to ($2p_{3/2}$, $3d$-t_{2g}) and ($2p_{3/2}$,$3d$-e_g) states while the second doublet ($2p_{1/2}$) (462-468 eV) originates from transitions to the corresponding $2p_{1/2}$ states. The $2p_{3/2} - 2p_{1/2}$ splitting is due to spin-orbit coupling while the t_{2g}-e_g separation is the crystal-field splitting due to the surrounding O atoms. [8,9] It has to be noted that in all spectra, the ($2p_{3/2}$, e_g) peak is broader than the ($2p_{3/2}$, t_{2g}) due to the large degree of hybridization of e_g orbitals with O ligand orbitals. [10] Satellite peaks at 470.5 and 476.0 eV due to polaronic transitions are also often observed. [11,12] The O 1*s* spectra (Fig. 3d, main manuscript) can be divided in two regions. The doublet between 528 and 536 eV can be attributed to O 1*s* excitation to hybrid excited states in which the final level is a mixture of O 2*p* and Ti 3*d* orbitals. The spectral features at 531.3 and 533.4 eV are assigned to the t_{2g} and e_g orbitals, respectively. [13] This region is very sensitive to local symmetry and coordination. Peaks in the region between 536 eV and 555 eV corresponds to O 1*s* excited states in which the final level is a hybridization of O 2*p* and Ti 4*sp* orbitals.[14] This region is more sensitive to long-range order.[13]

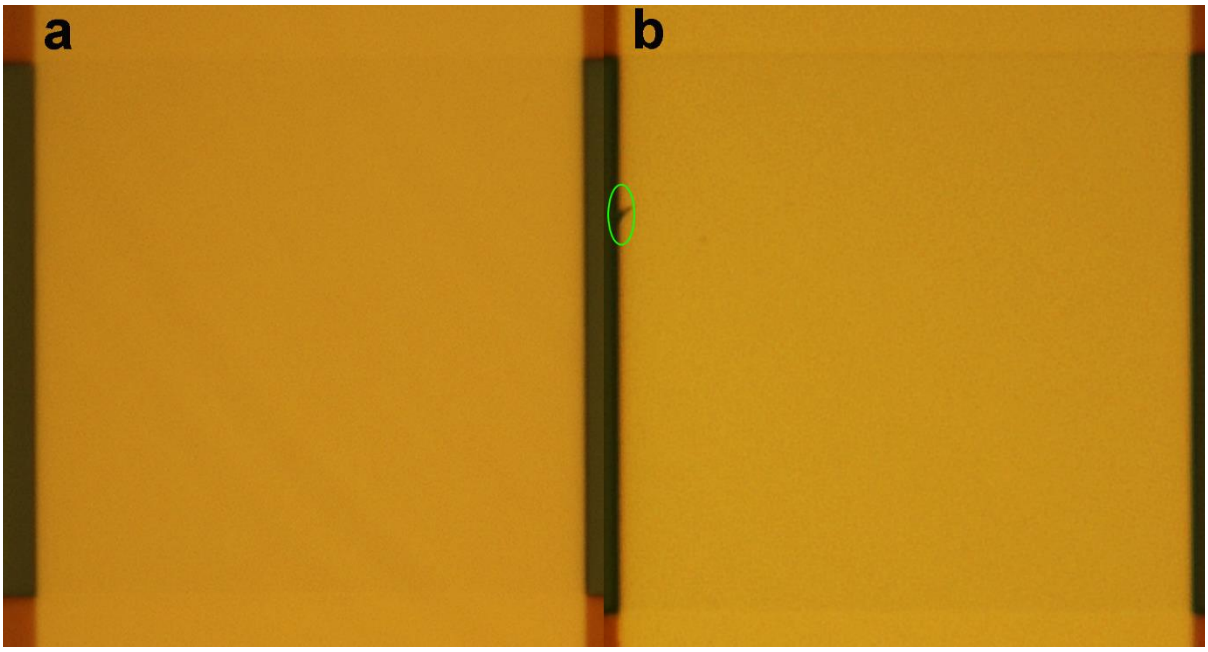

Figure S2 | Optical images of the device before **(a)** and after **(b)** switching into LRS. Morphological defect of TE is circled in green.

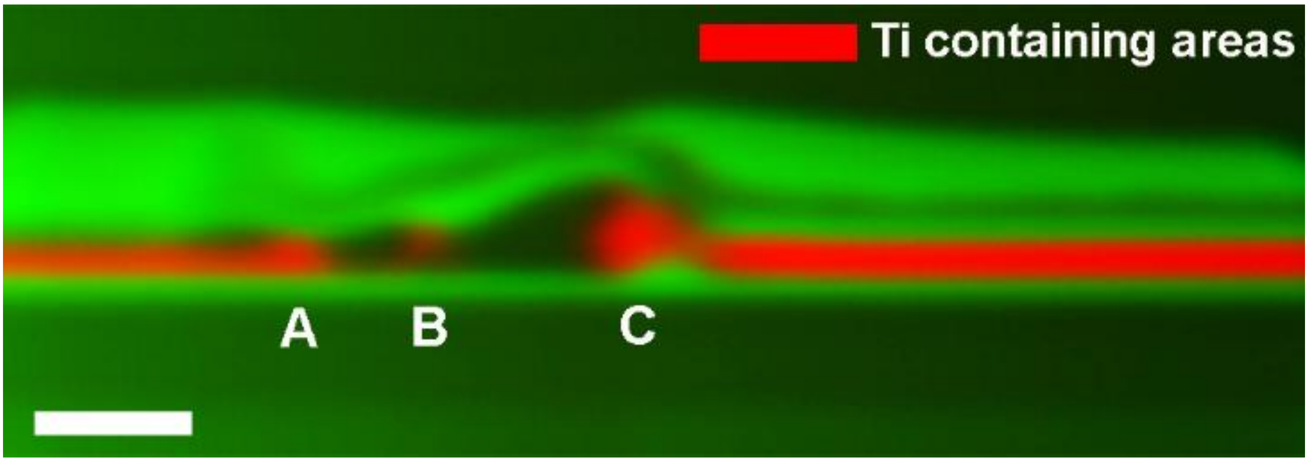

Figure S3 | Average of all images of the Ti stack showing in red the regions containing only Ti species. Scale bar = 200 nm.

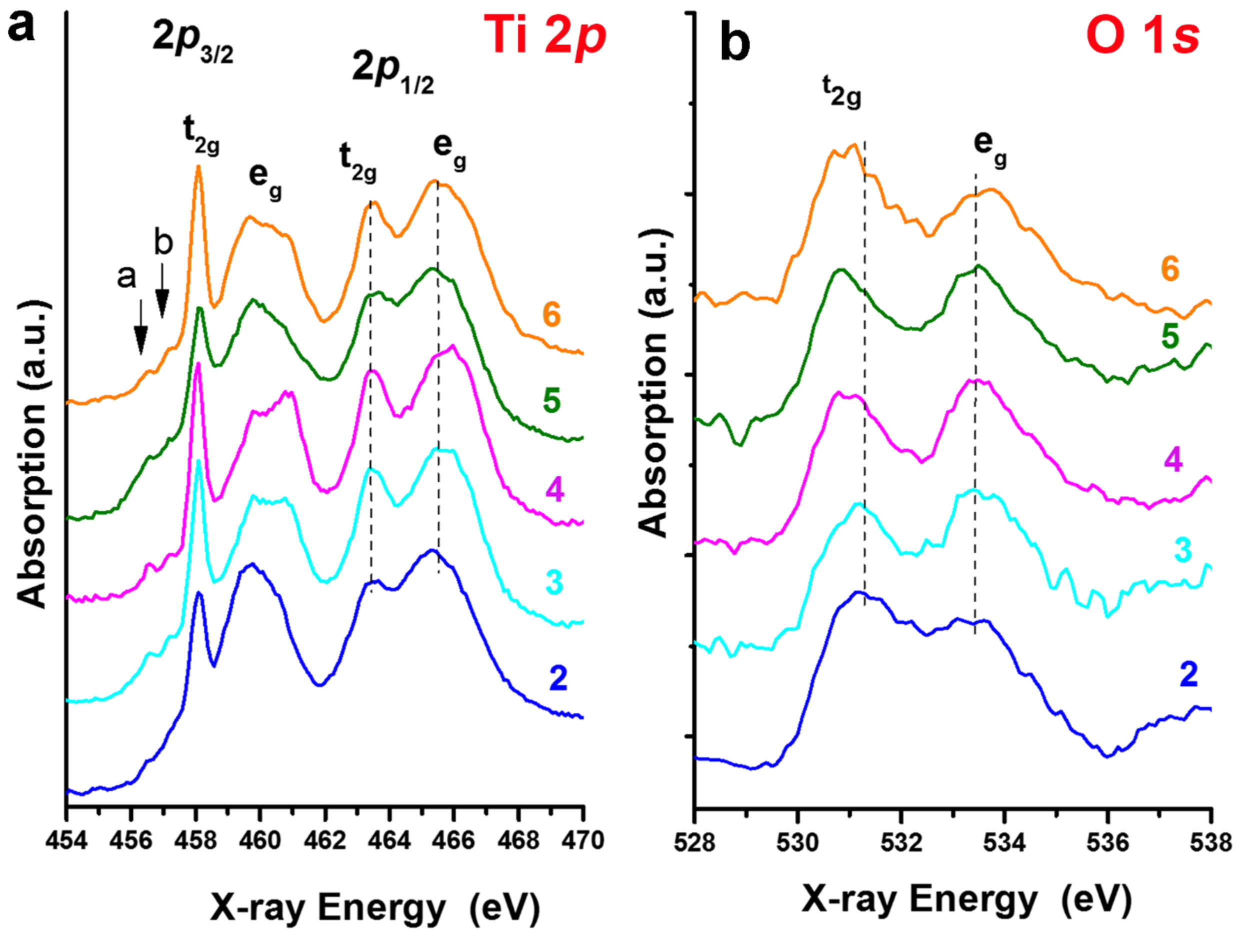

Figure S4 | NEXAFS point Ti 2*p* (a) and O 1*s* (b) spectra extracted from the ROI_2, ROI_3, ROI_4, ROI_5 and ROI_6 circled in the X-ray image of Fig. 5c in the main manuscript.

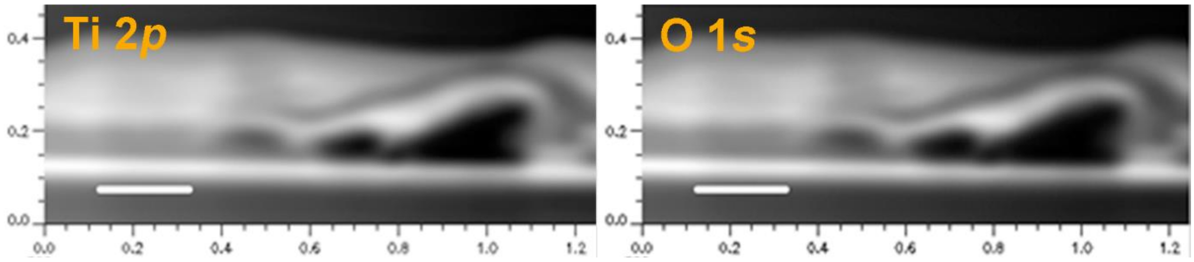

Figure S5 | Ti 2*p* and O 1*s* stacks adjusted to the same spatial area and mesh size corresponding to 4.75 nm pixel size, used for combined stack of Ti 2*p* and O 1*s*. The final size for both stack is 291x93 pixels. Scale bar = 200 nm.

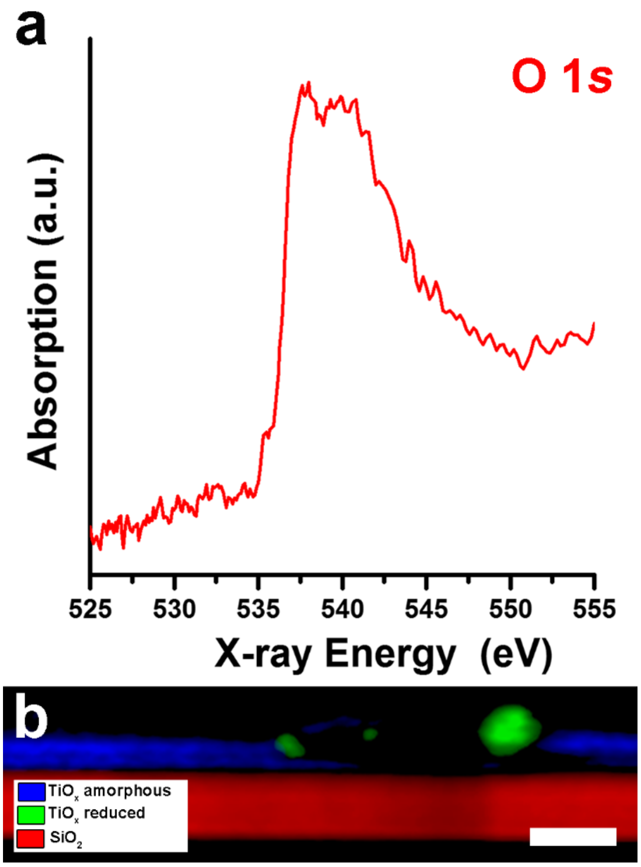

Figure S6 | (a) O 1*s* spectrum from the SiO_2 layer support. **(b)** Color-coded composition map of selected components: red (SiO_2), green (reduced TiO_x) and blue (amorphous TiO_x). Scale bar = 100 nm.

3. Chemical mapping

X-ray microscopy image sequences can be analysed to provide quantitative maps of chemical components [15, 16]. In this study maps of three spectrally distinct components - TiO_x amorphous, TiO_x reduced and a third component similar to TiO_x reduced but slightly different at the O 1s – were generated by fitting to linear combinations of reference spectra extracted from specific regions of the area measured. If additional TiO_x components were added, the component maps from the fit contained large regions with unphysical negative coefficients, indicative of an over-determined fit. If any one of the three reference spectra were removed from the fit, the residual of the fit increased significantly in the regions of the missing component. Figure S7 presents the reference spectra and color coded composite maps for the 3-component fit using stack fit analysis (which also includes a constant to fit the non-Ti, non-O components such as the Pt electrodes) to the separate (Ti 2*p* or O 1*s*) and the combined (Ti 2*p* appended O 1*s*, aligned) image sequences.

In order to derive quantitative thickness scales for component maps the normal procedure would be to scale the as-extracted reference spectra to the elemental response for a material of the expected elemental stoichiometry and density [15, 16] evaluated from tabulated X-ray absorption data [17], in which case the grey scales of the derived component maps would give nm thicknesses. However, because we do not know the composition or density, in this case the edge jumps in background subtracted Ti 2*p* and O 1*s* spectra were used to derived amounts of Ti and O based on the scaling to the corresponding edge jumps in pure elemental reference spectra of Ti ($\Delta_{480\text{-}450eV}$ = 0.0023) and O ($\Delta_{555\text{-}428eV}$ =0.0019) at a density of 1.0 g.cm^{-3}. [17] While this approach has quantitative uncertainties with respect to density, it does have the merit of giving a reliable measure of the O/Ti ratio since the density in any specific compositional region will be constant. This method was used to derive the O/Ti ratios reported in Table S1. All data manipulations were performed using aXis2000. [18] The edge jump values measured from the extracted X-ray absorption spectra have a precision of ~20 %, based on variability of the choice of 'reasonable' background subtraction.

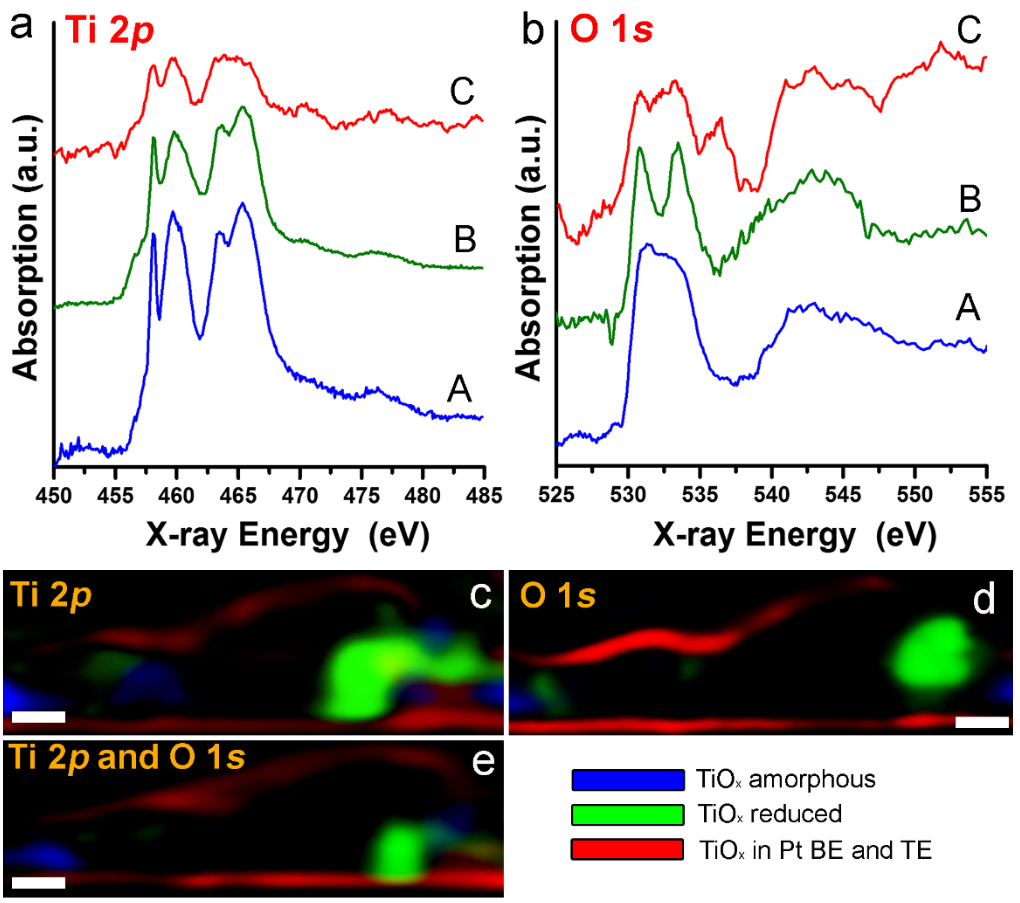

Figure S7 | NEXAFS Ti 2*p* **(a)** and O 1*s* **(b)** spectra of amorphous TiO_x (A), reduced TiO_x (B) and the Ti containing phase observed in the TE and BE (C). **(c-e)** Color-coded composition maps of selected components at the Ti 2*p* and O 1*s* generated independently **(c, d)** and combining Ti 2*p* and O 1*s* **(e)**. Blue (amorphous TiO_x), green (reduced TiO_x) and red (phase in Pt electrodes). Scale bar = 50 nm.

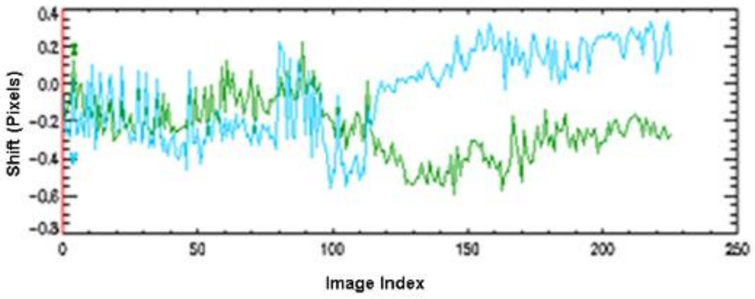

Figure S8 | Alignment of images using cross correlation. Shift in X direction (green line) and Y direction (cyano line) is less than ±0.6 pixels (± 3 nm).

	Amorphous TiO_x			Reduced TiO_x			Phase in TE/BE		
	Jump	*Thickness (nm)*	*O/Ti*	*Jump*	*Thickness (nm)*	*O/Ti*	*Jump*	*Thickness (nm)*	*O/Ti*
Ti	0.17	74±5	1.85±0.15	0.09	40±5	1.37±0.15	0.06	24±4	5±1
O	0.26	137±8		0.10	55±5		0.22	116±6	

Table S1. Edge jumps, absolute thickness of OD maps and O/Ti nominal ratio of amorphous and reduced TiO_x and third phase in TE and BE. Error were estimated from reproducibility with small variations in reference spectra.

References

1. Okada, K., Uozumi, T. & Kotani, A. Split-Off State Formation in the Final State of Photoemission in Ti Compounds. *J. Phys. Soc. Japan* **63,** 3176–3184 (1994).

2. Park, S.-J. *et al.* In situ control of oxygen vacancies in TiO_2 by atomic layer deposition for resistive switching devices. *Nanotechnology* **24,** 295202 (2013).

3. Strachan, J. P. *et al.* Direct identification of the conducting channels in a functioning memristive device. *Adv. Mater.* **22,** 3573–3577 (2010).

4. Strachan, J. P. *et al.* Characterization of electroforming-free titanium dioxide memristors. *Beilstein J. Nanotechnol.* **4,** 467–473 (2013).

5. Strachan, J. P. *et al.* Structural and chemical characterization of TiO_2 memristive devices by spatially-resolved NEXAFS. *Nanotechnology* **20,** 485701 (2009).

6. Borghetti, J. *et al.* Electrical transport and thermometry of electroformed titanium dioxide memristive switches. *J. Appl. Phys.* **106,** 124504 (2009).

7. Koehl, a. *et al.* Evidence for multifilamentary valence changes in resistive switching $SrTiO_3$ devices detected by transmission X-ray microscopy. *APL Mater.* **1,** 042102 (2013).

8. Sánchez-Santolino, G. *et al.* Characterization of surface metallic states in $SrTiO_3$ by means of aberration corrected electron microscopy. *Ultramicroscopy* **127,** 109–113 (2013).

9. Stoyanov, E., Langenhorst, F. & Steinle-Neumann, G. The effect of valence state and site geometry on Ti $L_{3,2}$ and O K electron energy-loss spectra of Ti_xO_y phases. *Am. Mineral.* **92,** 577–586 (2007).

10. Kucheyev, S. *et al.* Electronic structure of titania aerogels from soft x-ray absorption spectroscopy. *Phys. Rev. B* **69,** 245102 (2004).

11. Das, C., Tallarida, M., Schmeißer, D. Linear dichroism in ALD layers of TiO_2. *Environ. Earth Sci.* **70,** 3785–3795 (2013).

12. Laan, G. Van Der. Polaronic satellites in x-ray-absorption spectra. *Phys. Rev. B* **41,** 12366–12368 (1990).

13. Lusvardi, V. S. *et al.* An NEXAFS investigation of the reduction and reoxidation of TiO2(001). *Surf. Sci.* **397,** 237–250 (1998).

14. Chen, X. *et al.* Properties of disorder-engineered black titanium dioxide nanoparticles through hydrogenation. *Sci. Rep.* **3,** 1510 (2013).

15. Hitchcock, A. P. *Soft X-ray Imaging Spectromicroscopy, Handb. Nanoscopy* [Van Tendeloo, Van Dyck, D. and Pennycook, S. J. (ed.)] [745–791] (Wiley, 2012).

16. Ade, H. & Hitchcock, A. P. NEXAFS microscopy and resonant scattering : Composition and orientation probed in real and reciprocal space. *Polymer.* **49,** 643–675 (2008).

17. Henkel; B.L., Gullikson; E.M. & Davis; J.C. X-ray interactions: photoabsorption, scattering, transmission, and reflection at E=50-30000 eV, Z=1-92. *At. Data Nucl. Data Tables* **54,** 181–342 (1993).

18. Hitchcock, A. *aXis2000. Available Free noncommercial use from http//unicorn.mcmaster.ca/aXis2000.html* at <http://unicorn.mcmaster.ca/aXis2000.html>
